# Effectiveness and safety of tenofovir alafenamide in children and adolescents living with HIV: a systematic review

**DOI:** 10.1002/jia2.26037

**Published:** 2023-02-23

**Authors:** John O'Rourke, Claire L. Townsend, Edith Milanzi, Intira Jeannie Collins, Hannah Castro, Ali Judd, Marissa Vicari, Julie Jesson, Valériane Leroy, Martina Penazzato, Françoise Renaud

**Affiliations:** ^1^ Consultants to the World Health Organization Geneva Switzerland; ^2^ International AIDS Society Geneva Switzerland; ^3^ MRC Clinical Trials Unit at UCL University College London London UK; ^4^ Centre for Epidemiology and Research in POPulation Health (CERPOP) Inserm, Université de Toulouse Université Paul Sabatier Toulouse France; ^5^ HIV Department World Health Organization Geneva Switzerland

**Keywords:** children, drug‐related side effects and adverse reactions, HIV, systematic review, tenofovir, treatment outcome

## Abstract

**Introduction:**

Tenofovir alafenamide (TAF) is approved for paediatric use in fixed‐dose combination tablets, but efficacy and safety data in children are limited. We conducted a systematic review on the efficacy/effectiveness and safety of TAF in infants, children and adolescents living with HIV.

**Methods:**

We searched MEDLINE, Embase, the Cochrane Library, clinical trial registries, reference lists and relevant conferences to identify literature published January 2009–March 2021. We included clinical trials and observational studies assessing the efficacy/effectiveness or safety of TAF through ≥6 months of treatment in participants aged 0–19 years.

**Results and discussion:**

Overall 3626 abstracts and 371 full papers were screened. Four single‐arm, innovator‐funded trials (341 participants) and a pooled analysis of those trials were identified. All four trials included treatment‐experienced and virally suppressed children or adolescents. One trial also included treatment‐naïve adolescents with baseline viral load >1000 copies/ml. The risk of bias was rated as low in one study and unclear in the other three owing to missing data on study design (all conference presentations). At 48 weeks, 92% (46/50) of treatment‐naïve participants were virally suppressed (one trial). Among treatment‐experienced participants with viral load at 48 weeks, 214 of 224 participants were virally suppressed. Across the studies, one grade 3/4 adverse event was considered drug‐related (intermediate uveitis). There were three discontinuations for adverse events (grade 2 anxiety and insomnia, grade 1 iridocyclitis [drug‐related] and grade 1 pulmonary tuberculosis [unrelated to treatment]). One accidental death occurred across the four studies. In the pooled analysis of 223 participants, the median change in bone mineral density *z*‐score (height‐ and age‐adjusted) from baseline to 48 weeks was −0.12 (interquartile range [IQR] −0.46, 0.17) to 0.05 (IQR not reported) for spine, and −0.09 (IQR −0.33, 0.07) to 0.09 (IQR not reported) for total body less head. Weight‐for‐age *z*‐scores increased by 0.25 from baseline to 48 weeks.

**Conclusions:**

Four single‐arm trials were identified in this systematic review, with initial evidence suggesting good viral suppression and no obvious safety concerns in children and adolescents on TAF‐containing regimens over 24–48 weeks. However, further comparative and longer‐term safety data are needed in children and adolescents, including on weight and metabolic changes.

## 1 INTRODUCTION

Tenofovir alafenamide (TAF) is an oral prodrug of tenofovir, a nucleoside reverse transcriptase inhibitor (NRTI), that is closely related to tenofovir disoproxil fumarate (TDF). TAF produces higher intracellular and lower plasma concentrations of tenofovir than TDF and can, therefore, be administered at a lower dose (25 mg once daily for adults vs. 300 mg for TDF) [1]. The use of pharmacokinetic boosters, such as ritonavir or cobicistat, increases plasma concentrations of tenofovir, and daily doses of TAF are, therefore, adjusted down (from 25 to 10 mg) to account for this boosting effect [2]. TAF was approved for use in adults living with HIV in 2016. A systematic review updated in 2020 found no difference in efficacy and safety between TDF and TAF in adults when used without a booster [2, 3], but boosted TDF was associated with lower efficacy and an increased risk of discontinuation due to renal events compared with boosted TAF, possibly due to differences in dosing and plasma concentrations [3]. However, there have been reports of increases in body weight and lipid levels in adults taking TAF [4, 5, 6, 7], and the long‐term safety of TAF is unknown. In this context, the World Health Organization (WHO) continues to recommend TDF as the preferred antiretroviral drug in first‐line therapy in adults (combined with dolutegravir and lamivudine or emtricitabine), with TAF considered a favourable option for special circumstances, when bone and renal toxicity are a particular concern [8].

TDF has been widely used in adolescents and adults since 2001 and was approved for use in children from 2 years of age in 2012. However, there are concerns about loss of bone mineral density (BMD) in children on TDF, since children have higher bone turnover than adults due to skeletal growth [9, 10]. Abacavir is, therefore, considered the preferred NRTI in infants and children [11, 12], but there have been increasing concerns about the selection of abacavir resistance as a result of maternal antiretroviral therapy (ART) exposure or failure of first‐line regimens [8, 13]. There is, therefore, a potential role for TAF as an alternative NRTI in children for first‐ or second‐line treatment. For these reasons, TAF‐based paediatric formulations have been highlighted as priority agents for public health use since 2016 [14, 15].

TAF is approved for use in treating ART‐naïve and ‐experienced children and adolescents weighing at least 25 kg as part of fixed‐dose combination tablets co‐formulated with either bictegravir and emtricitabine (B/F/TAF; since 2019) or elvitegravir, cobicistat and emtricitabine (EVG/COBI/FTC/TAF, since 2017), or with emtricitabine alone (F/TAF, since 2017). Since 2019, WHO has recommended TAF in children as an alternative first‐line drug in combination with lamivudine (or emtricitabine) and dolutegravir for age and weight groups with approved TAF dosing [11]. A low‐dose single‐tablet formulation of B/F/TAF was approved in October 2021 by the United States Food and Drug Administration for use in children from 14 kg. However, limited data are available on the use of TAF in younger children, and medium‐ to long‐term safety in the paediatric population remains unknown. We, therefore, conducted a systematic review on the efficacy/effectiveness and safety of TAF in infants, children and adolescents, to inform the WHO HIV treatment guidelines updates [8] as well as the paediatric antiretroviral drug optimization process [15].

## 2 METHODS

This systematic review followed the Centre for Reviews and Dissemination guidance [16], and the protocol was published in the PROSPERO International Prospective Register of systematic reviews (registration number CRD42020204432) [17]. Results are reported according to Preferred Reporting Items for Systematic Reviews and Meta‐Analyses (PRISMA) guidelines [18]. The full review assessed five antiretroviral drugs (dolutegravir, raltegravir, darunavir, lopinavir and TAF), and results relating to TAF are reported here. Records were managed in Endnote X9 and Microsoft Excel.

### 2.1 Data sources and search strategies

We searched MEDLINE, MEDLINE In‐Process, MEDLINE E‐pub ahead of print (via Ovid), Embase (via Ovid) and the Cochrane Library using free‐text terms and index terms (File S1). We also searched abstract databases of selected conferences occurring between 2018 and March 2021. Reference lists from key HIV treatment guidelines and from all included papers were screened, and clinical trial registries were searched to identify ongoing studies.

We screened English‐ and French‐language publications reporting on the use of TAF in treatment‐naïve or ‐experienced infants, children and/or adolescents aged 0–19 years living with HIV (Table 1). Observational studies and clinical trials published 1 January 2009–21 March 2021 were included, to cover the period when TAF and other drugs included in the review were approved for use in children and adolescents. Studies were eligible if they reported efficacy or effectiveness and/or safety outcomes in children or adolescents on TAF followed up for at least 6 months (Table 1). Allowances were made for studies reporting outcomes at approximately 6 months (e.g. at 24 weeks). We excluded case studies, reviews and letters to the editor, studies reporting outcomes in infants exposed to HIV *in utero* or through breastfeeding and studies reporting only pharmacokinetic data or drug–drug interactions. We also excluded studies reporting only on adults aged ≥18 years or pregnant women.

**Table 1:** Eligibility criteria

PICO criteria	Description
Population	Children and adolescents (aged 0–19 years^a^) living with HIV who were treatment‐naïve or treatment‐experienced
Intervention^b^	Tenofovir alafenamide in combination with other antiretroviral therapies recommended for use in a paediatric or adolescent population
Comparator	Dolutegravir, raltegravir, darunavir, lopinavir, or no comparator (i.e. single‐arm trials were included)^b^
Primary outcomes	Efficacy/effectiveness measured through 6 months follow‐up or more CD4 cell counts or percent (change from baseline) HIV‐1 RNA viral load Safety measured through 6 months follow‐up or more Mortality Treatment discontinuation or change and reasons Grade 3/4 adverse events and their association with antiretroviral drugs
Secondary outcomes	Safety measured through 6 months follow‐up or more Hospitalization Growth and weight gain Diabetes Bone health Renal function Hypersensitivity reactions Lipid levels (cholesterol and triglycerides; change from baseline)
Study design	Observational studies or clinical trials (case studies were excluded)
Publication period	Published between 1 January 2009 and date of searches^c^
Language	English and French language publications

Abbreviation: PICO, population, intervention, comparator, outcome.

^a^ Studies reporting on an adult population that included participants aged ≥18 years only were excluded, as were studies assessing the effect of exposure to antiretroviral drugs in pregnancy or postnatally.

^b^ Regardless of the comparator arm, only data from the intervention listed were extracted.

^c^ Searches were initially run in August 2020 and updated in March 2021.

### 2.2 Article screening and data extraction

All abstracts and papers were screened independently, in duplicate, by three reviewers (JOR, CT and EM) against the eligibility criteria, and decisions were recorded in a Microsoft Excel spreadsheet. Any disagreements were discussed between the reviewers and resolved with another member of the project team if necessary. Data from all selected articles were extracted into a standard Microsoft Excel form by one reviewer and independently checked by another reviewer. The risk of bias was assessed independently by two reviewers using the Cochrane Risk of Bias Tool (Version 2) for randomized controlled trials, the National Institute of Health quality assessment tool for non‐randomized interventional studies and the CLARITY tool for cohort studies.

The following data were extracted: study characteristics (design, country, time period, duration of follow‐up, inclusion criteria and number of participants); participant characteristics (age, sex, treatment experience, baseline viral load and CD4 cell count/percent); efficacy/effectiveness and safety data (File S1). Primary efficacy/effectiveness outcomes were HIV RNA viral load and change in CD4 counts. Primary safety outcomes were mortality, grade 3/4 adverse events [19] and treatment discontinuation. Secondary outcomes included hypersensitivity reactions, growth, and measures of bone health and renal function. For binary and categorical variables, we extracted the number and proportion of participants with an event. For studies reporting interim results for viral load outcomes, the denominator used was the number of participants with data available at that time point. For continuous variables, we extracted the mean, median, range, interquartile range (IQR), change from baseline, regression coefficients and 95% confidence intervals where available. Data that were only reported in a figure were extracted using the online tool “Web Plot Digitizer” [20].

### 2.3 Definitions

Study populations were defined as infants, children and adolescents if the participants were aged <12 months, 1 to <12 years and 12–19 years, respectively. Standard definitions for grade 3 (severe) and grade 4 (potentially life‐threatening) adverse events were used and included clinical signs and symptoms and laboratory findings [19]. The number of participants who experienced at least one grade 3 or 4 adverse events was extracted, rather than the total number of events occurring during the study. We did not extract grade 1 or grade 2 adverse events, or “serious adverse events,” unless the grade of event was reported.

### 2.4 Evidence synthesis

The results of the systematic review were synthesized narratively; no meta‐analyses were undertaken. Results for viral load are presented as bar charts, and data on discontinuations, adverse events and mortality are presented in a heatmap; both were generated in Microsoft Excel.

### 2.5 Deviations from the protocol

Slight modifications were made to the protocol following its publication on PROSPERO. An additional risk of bias tool was added to assess non‐randomized interventional studies (discussed above). An additional outcome of interest—changes in cholesterol and triglycerides from baseline—was also added.

## 3 RESULTS AND DISCUSSION

### 3.1 Main results

#### 3.1.1 Study selection

**Figure 1:**
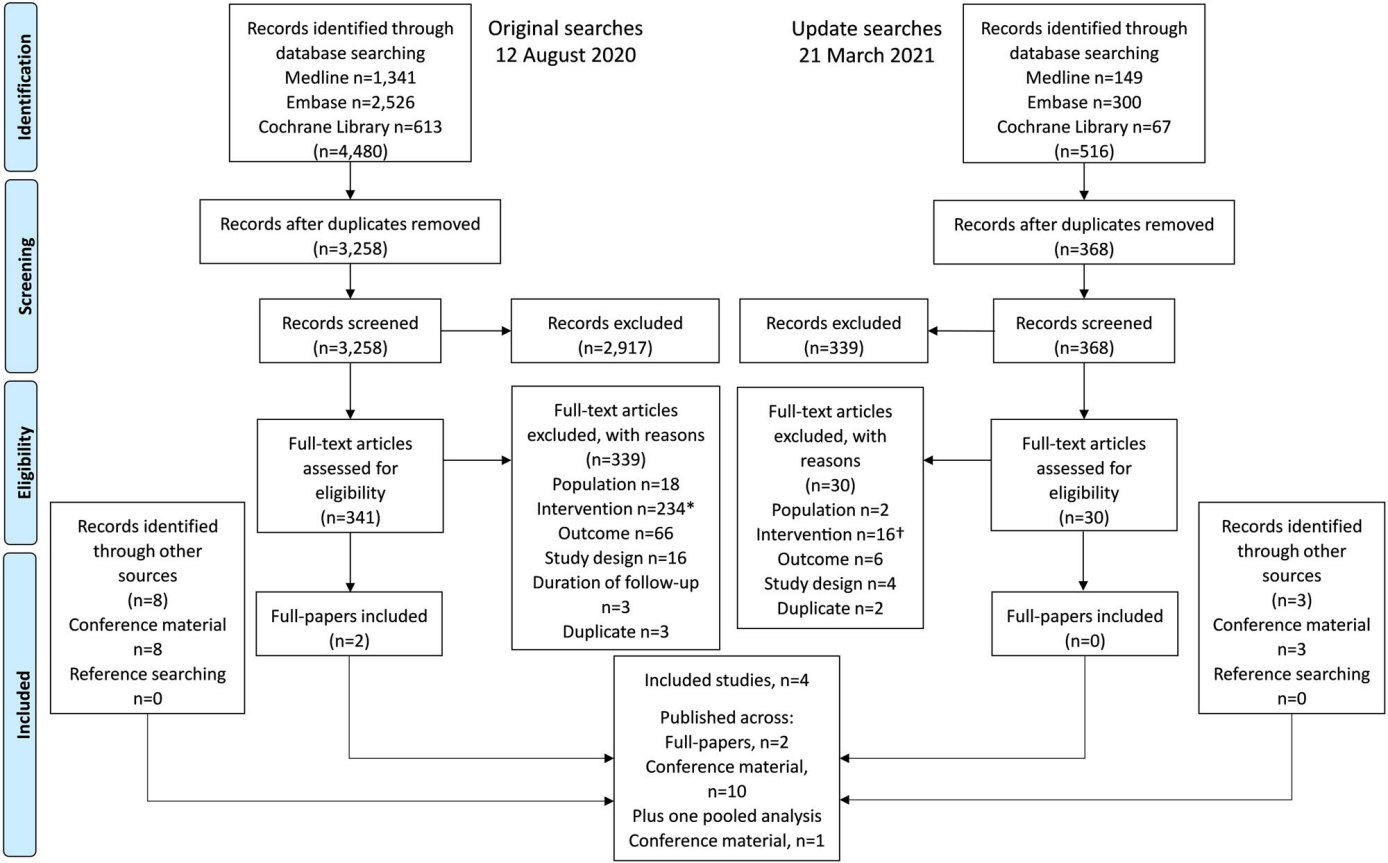
PRISMA flow diagram illustrating the number of included and excluded studies at each stage of the systematic review. Note: The search strategies included terms for dolutegravir, raltegravir, darunavir, and lopinavir (as well as TAF); however, during full paper review, studies were categorized by the treatment of interest and only studies assessing TAF are included in this publication. * Of the 234 publications excluded because they assessed an intervention other than TAF, 15 full papers reported data on dolutegravir or raltegravir, and 34 full papers reported on darunavir or lopinavir (solid formulations). † Of the 16 publications excluded because they assessed an intervention other than TAF, two full papers reported data on dolutegravir or raltegravir, and six reported on darunavir or lopinavir (solid formulations).

The searches were carried out on 12 August 2020 and updated on 21 March 2021 to capture any additional publications. The number of studies identified at each stage of the review is shown in a PRISMA flow diagram (Figure 1). A total of 3626 abstracts and 371 full papers were screened. Eleven conference abstracts and two full papers relating to four trials and one pooled analysis of a subset of participants of those four trials assessing TAF in children and/or adolescents were retrieved (GS‐US‐292‐0106, GS‐US‐292‐1515, GS‐US‐380‐1474 and GS‐US‐311‐1269) [21, 22, 23, 24, 25, 26, 27, 28, 29, 30, 31, 32, 33]. In addition, two ongoing trials were identified through searches of clinical trial registries (CHAPAS‐4 and GS‐US‐216‐0128) [34, 35]. No study results were available for these ongoing trials, but study characteristics are presented in File S2 (Table S1). Estimated completion dates were reported to be February 2023 (CHAPAS‐4) and April 2026 (GS‐US‐216‐0128).

#### 3.1.2 Study and participant characteristics

The four trials were non‐randomized, single‐arm, open‐label and innovator company‐funded, and included a total of 341 children and adolescents. The pooled analysis reported only on weight change and lipids over 48 weeks in a subset of participants enrolled in the four trials (*n* = 223) [33]. The studies all took place across multiple countries, which included South Africa (*n* = 4), the United States of America (*n* = 4), Thailand (*n* = 2), Uganda (*n* = 2), Zimbabwe (*n* = 1) and Panama (*n* = 1) (Table 2). Baseline characteristics for the 341 participants included across the four studies are shown in Table 2. Three of the studies included children and adolescents (aged 2 to <18 years), and one reported only on adolescents (aged 12 to <18 years) (GS‐US‐292‐1515) (Table 2) [26]. All four studies included participants who were virally suppressed (<50 copies/ml), with CD4 counts ≥100 or ≥200 cells/μl and on a stable regimen at baseline (291/341, 85.3%) (referred to as “treatment‐experienced” in this review) (Table 2). One study (GS‐US‐292‐0106) also enrolled treatment‐naïve adolescents with baseline HIV RNA viral load >1000 copies/ml (50/341, 14.7%; Table 2) [21]. There were no studies in children aged <2 years. Two studies assessed the single‐tablet, fixed‐dose combination of EVG/COBI/FTC/TAF (GS‐US‐292‐0106 and GS‐US‐292‐1515) (boosted regimen) [21, 22, 23, 24, 25, 26]; one study assessed the single‐tablet combination of B/F/TAF (unboosted regimen) (GS‐US‐380‐1474) [25, 27, 28, 29, 30], and one assessed the fixed‐dose combination of F/TAF taken with a boosted or unboosted third antiretroviral drug (ritonavir‐boosted lopinavir or ritonavir‐boosted atazanavir for children aged 2 to <12 years; ritonavir‐boosted lopinavir, ritonavir‐boosted darunavir or cobicistat‐boosted darunavir for children aged 6 to <12 years; ritonavir‐boosted lopinavir or efavirenz for adolescents aged 12 to <18 years) (GS‐US‐311‐1269) [31, 32]. Duration of follow‐up was at least 48 weeks for all sub‐groups of children and adolescents except the adolescent cohort of GS‐US‐311‐1269 (F/TAF + efavirenz or ritonavir‐boosted lopinavir), for which only data at 24 weeks were available (Table 2) [31].

**Table 2:** Study and participant characteristics of the four single‐arm trials and one pooled analysis identified in the systematic review of tenofovir alafenamide in children and adolescents

Study (phase)	Study refs	Country	Population	Intervention (formulation and dose of TAF)	Study duration (weeks)	*N*	Median age^a^ (range; years)	Treatment history	Viral suppression; CD4 counts at baseline	Available outcome data
GS‐US‐292‐0106 (Phase 2/3)	[24, 25]	South Africa, Uganda, Zimbabwe, USA and Thailand	Children (≥2 years; ≥14 to <25 kg)	EVG/COBI/FTC/TAF (6 mg QD as part of single tablet containing all four ARVs)	48	27	6 (3, 9)	Experienced	Yes; unclear	VL, mortality, grade 3/4 AE, drug‐related grade 3/4 AE, discontinuation due to AE, growth, bone health, renal health
	[22, 23]	Uganda, USA and Thailand	Children (6 to <12 years; ≥25 kg)^b^	EVG/COBI/FTC/TAF (10 mg QD in capsule shaped single tablet containing all four ARVs)	96	52	10 (7, 11)	Experienced	Yes; ≥100 cells/μl	VL, grade 3/4 AE, discontinuation due to AE, bone health, renal health
	[21]	South Africa, Thailand, Uganda and the USA	Adolescents (12 to <18 years; ≥35 kg)^b^	EVG/COBI/FTC/TAF (10 mg QD as part of single tablet containing all four ARVs)	48	50	15 (12, 17)	Naïve	No; ≥200 cells/μl	VL, mortality, grade 3/4 AE, drug‐related grade 3/4 AE, discontinuation due to AE, growth, bone health, renal health
GS‐US‐292‐1515	[26]	South Africa and the USA	Adolescents (12 to <18 years; ≥35 kg)	EVG/COBI/FTC/TAF (10 mg QD as part of single tablet containing all four ARVs)	48	50	15 (12, 17)	Experienced	Yes; not specified	VL, mortality, drug‐related grade 3/4 AE, discontinuation due to AE, bone health, renal health
GS‐US‐380‐1474 (Phase 2/3)	[28]	South Africa, Thailand and the USA	Children (≥2 years; 14 to <25 kg)	B/F/TAF (low dose) (15 mg QD as part of single tablet containing all three ARVs)	48	22	6 (3, 9)	Experienced	Yes; ≥200 cells/μl	VL, mortality, grade 3/4 AE, drug‐related grade 3/4 AE, discontinuation due to AE, renal health
	[27, 29, 30]	South Africa, Thailand, Uganda and the USA	Children (6 to <12 years; ≥25 kg) and adolescents (12 to <18 years; ≥35 kg)	B/F/TAF (25 mg QD as part of single tablet containing all three ARVs)	48	100	10 (6, 11) 15 (12, 17)^c^	Experienced	Yes; ≥200 cells/μl	VL, mortality, clinical grade 3/4 AE, discontinuation due to AE, renal health
GS‐US‐311‐1269 (Phase 2/3)	[32]	Country not reported	Children (2 to <12 years; 17 to <25 kg)	F/TAF+(LPV/r or ATZ/r) (15 mg QD tablet)	48	3	8 (6, 8)	Experienced	Yes; ≥200 cells/μl	VL, mortality, grade 3/4 AE, drug‐related grade 3/4 AE, discontinuation due to AE, bone health, renal health
	[32]	Country not reported	Children (6 to <12 years; ≥25 kg)	F/TAF+(LPV/r, DRV/r or DRV/c) (25 mg QD tablet)	48	9	10 (8, 11)	Experienced	Yes; ≥200 cells/μl	VL, mortality, grade 3/4 AE, drug‐related grade 3/4 AE, discontinuation due to AE, bone health, renal health
	[31]	Panama, South Africa and the USA	Adolescents (12 to <18 years; ≥35 kg)	F/TAF+(LPV/r or EFV) (10 mg QD tablet + LPV/r; 25 mg QD tablet + EFV)	24	28	14 (12, 17)	Experienced	Yes; ≥200 cells/μl	VL, mortality, clinical grade 3/4 AE, drug‐related grade 3/4 AE, discontinuation due to AE, bone health, renal health
Rakhmanina 2020 (Pooled analysis)	[33]	Panama, South Africa, Thailand, Uganda and the USA	Children and adolescents (6 to <18 years)	TAF‐based regimen (QD)	48	223^d^	Mean: 12 (6, 17)	Naïve and experienced	Yes;see individual studies	Growth, weight gain

Abbreviations: AE, adverse event; ARVs, antiretroviral drugs; ATZ/r, ritonavir‐boosted atazanavir; B/F/TAF, bictegravir, emtricitabine and tenofovir alafenamide; DRV/c, cobicistat‐boosted darunavir; DRV/r, ritonavir‐boosted darunavir; EFV, efavirenz; EVG/COBI/FTC/TAF, elvitegravir, cobicistat, emtricitabine and tenofovir alafenamide; F/TAF, emtricitabine and tenofovir alafenamide; LPV/r, ritonavir‐boosted lopinavir; QD, once daily; refs, references; USA, United States of America; VL, viral load <50 copies/ml.

^a^ Median age unless otherwise specified.

^b^ Children (6 to <12 years) were followed‐up from July to September 2015; adolescents were followed‐up from May 2013 to August 2015. None of the other studies reported the time period that the study took place.

^c^ Median age of children (6 to <12 years; ≥25 kg) and adolescents (12 to <18 years; ≥35 kg), respectively.

^d^ Pooled analysis: all participants were also included in one of the four single‐arm trials.

#### 3.1.3 Risk of bias

GS‐US‐292‐0106 was the only study rated as having a low risk of bias and the only one with full published results (File S2, Figure S1) [21, 22]. The other three studies were reported as conference presentations, and limited details were provided on areas needed to assess bias; they were, therefore, rated as having unclear risk of bias. In two of these studies (GS‐US‐292‐1515 and GS‐US‐311‐1269) [26, 31, 32, 33], the number of eligible and enrolled children and adolescents was not given, and sample size calculations were not reported in any of the three studies.

#### 3.1.4 Effectiveness outcomes

**Figure 2:**
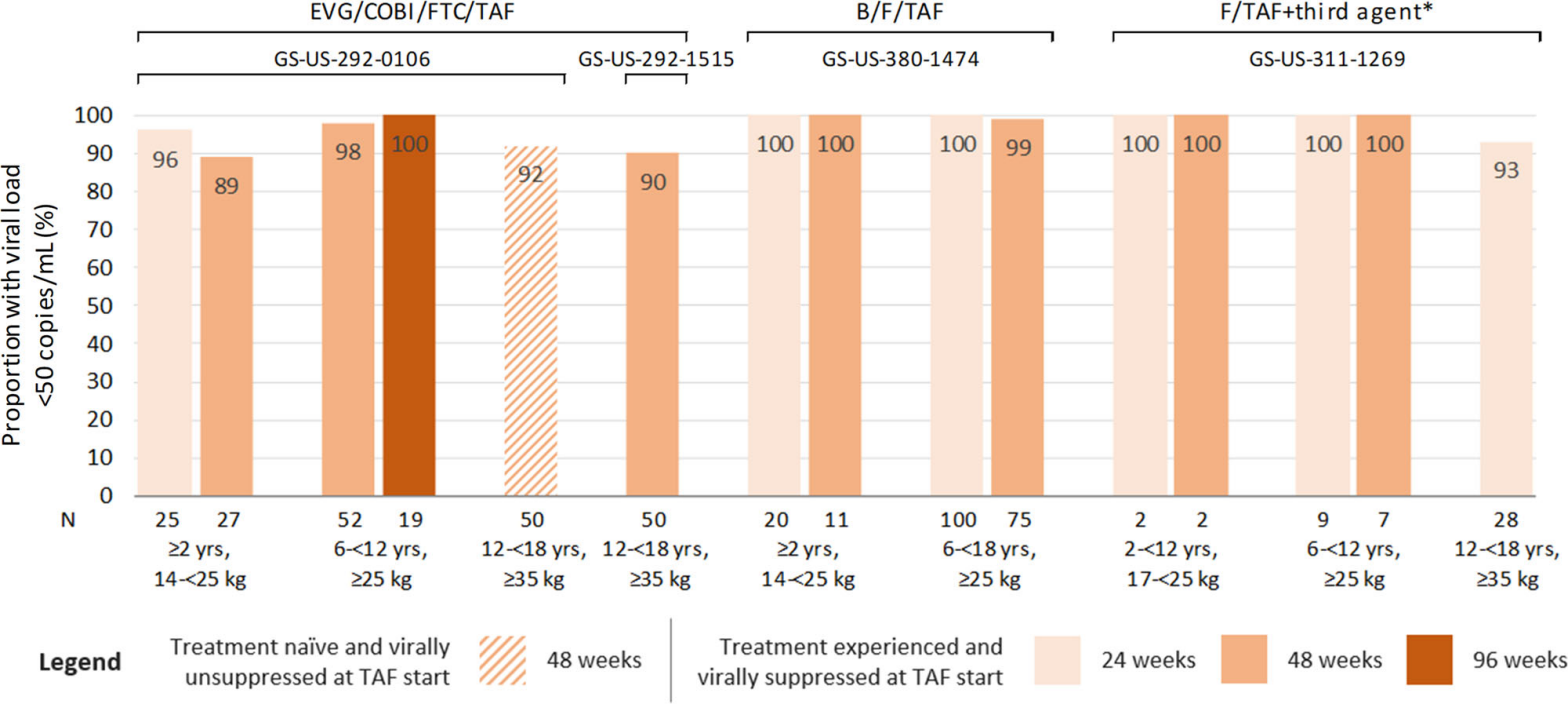
Proportion of participants with HIV viral load <50 copies/ml following treatment with TAF‐based regimens. The colour of the bars indicates the follow‐up time: 24 weeks (light orange), 48 weeks (medium orange) and 96 weeks (dark orange). * Third agent was ritonavir‐boosted lopinavir or ritonavir‐boosted atazanavir for children aged 2 to <12 years, ritonavir‐boosted lopinavir, ritonavir‐boosted darunavir, or cobicistat‐boosted darunavir for children aged 6 to <12 years, and ritonavir‐boosted lopinavir or efavirenz for adolescents aged 12 to <18 years. Abbreviations: B/F/TAF, bictegravir, emtricitabine and tenofovir alafenamide; EVG/COBI/FTC/TAF, elvitegravir, cobicistat, emtricitabine and tenofovir alafenamide; F/TAF, emtricitabine and tenofovir alafenamide.

Viral load data for children and adolescents were reported in the four trials, but not in the pooled analysis (Figure 2). In the one study reporting data on ART‐naïve adolescents with viral load >1000 copies/ml at baseline starting first‐line EVG/COBI/FTC/TAF, 92% (46/50) achieved HIV RNA viral load <50 copies/ml at 48 weeks (GS‐US‐292‐0106) [21]. Across the sub‐groups of treatment‐experienced children and adolescents, the proportion with viral suppression ranged from 93% (26/28) to 100% (20/20; 100/100; 2/2; 9/9) at 24 weeks and 89% (24/27) to 100% (11/11; 2/2; 7/7) at 48 weeks [23, 25, 26, 27, 28, 31, 32]. Overall, 214 of 224 treatment‐experienced participants were suppressed at 48 weeks follow‐up. One study had extended follow‐up to 96 weeks and reported that all the children with data available (19/19) had viral load <50 copies/ml [23]. Given that viral suppression was high across all included studies, no apparent difference in suppression was observed according to whether regimens were boosted or unboosted (Figure 2).

The most commonly reported CD4 outcome was the mean change in CD4 cell count. At 48 weeks, among treatment‐experienced children and adolescents, change in CD4 cell count from baseline ranged from −25.4 cells/μl (standard deviation [SD] 162, *n* = 100; median CD4 count at baseline >750 cells/μl) in children and adolescents receiving B/F/TAF, to 210 cells/μl (SD 407, *n* = 9; median CD4 count at baseline: 910 [IQR 689, 1133] cells/μl) in children aged 6 to <12 years old and ≥25 kg on F/TAF with ritonavir‐boosted lopinavir, ritonavir‐boosted darunavir or cobicistat‐boosted darunavir [25, 27, 32]. Change from baseline data were not reported for the 50 ART‐naïve adolescents receiving EVG/COBI/FTC/TAF, but the mean CD4 cell count was 685 cells/μl (SD 245.4) after 48 weeks of follow‐up compared with 471 cells/μl (SD 212.2) at baseline [21].

#### 3.1.5 Safety outcomes

**Figure 3:**
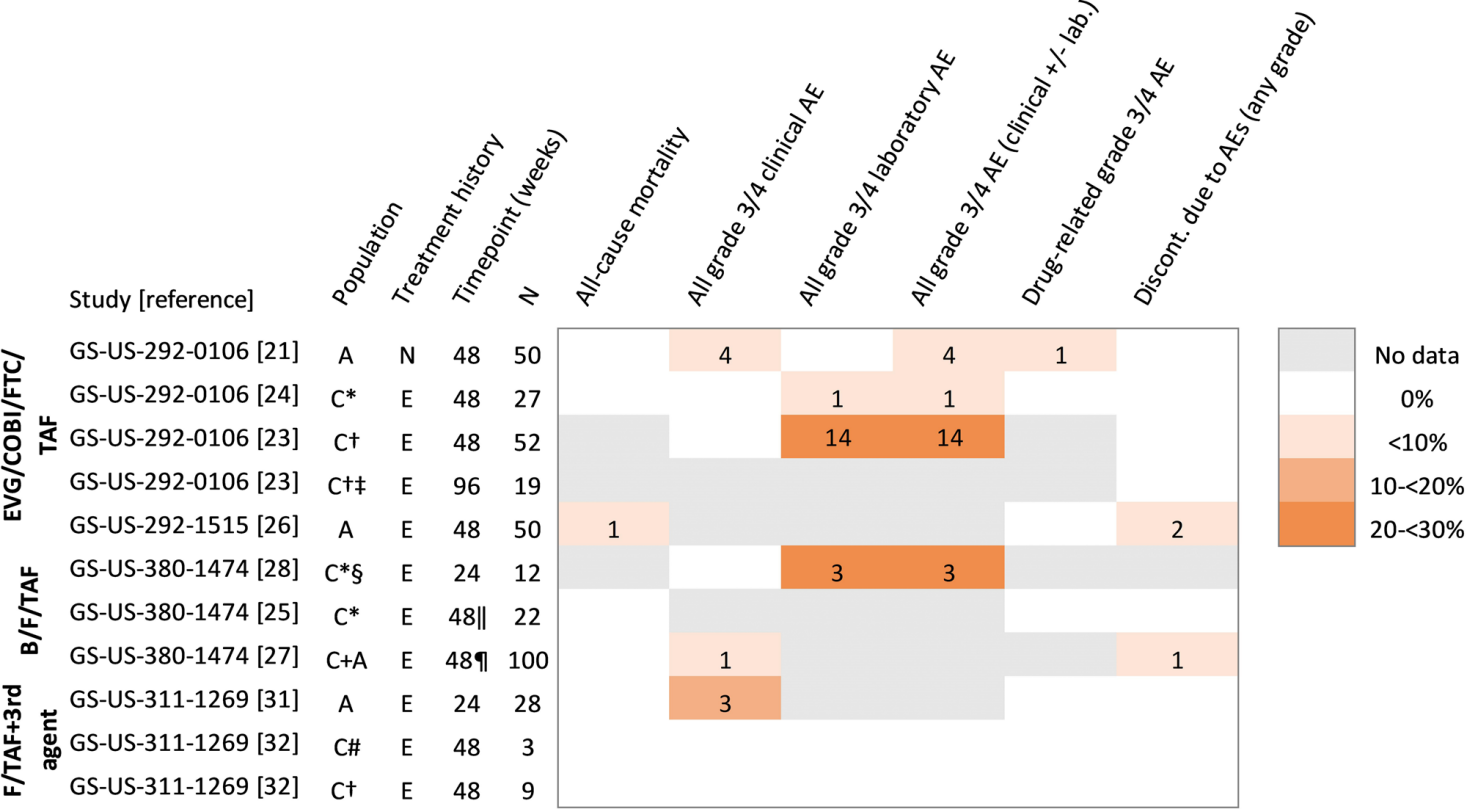
Proportion of participants on tenofovir alafenamide with reported outcomes in the four single‐arm trials identified in the systematic review of tenofovir alafenamide (January 2009–March 2021); ordered by study and time point at which outcomes were reported. Multiple rows for the same study correspond to different sub‐populations (age groups) and/or different time points. The numbers in the coloured cells correspond to the number of participants who experienced events. Discontinuations reported are for any adverse events regardless of grade. * ≥2 years of age and 14 to <25 kg; † 6 to <12 years of age and ≥25 kg; ‡ Subset of population with follow‐up to 96 weeks; § Interim analysis of children who had reached 24 weeks of follow‐up; ‖ 12 of 22 children had reached 48 weeks of follow‐up; median duration of exposure 54.9 weeks (interquartile range 29.3, 66.4); ¶ 76 of 100 participants had reached 48 weeks of follow‐up; # 2 to <12 years of age and 17 to <25 kg. Population groups: I, infants; C, children; A, adolescents. Treatment history: N, naïve; E, experienced at the start of TAF. Abbreviations: AE, adverse event; B/F/TAF, bictegravir, emtricitabine and tenofovir alafenamide; discont., discontinuation; EVG/COBI/FTC/TAF, elvitegravir, cobicistat, emtricitabine and tenofovir alafenamide; F/TAF+3rd agent, emtricitabine and tenofovir alafenamide, plus third agent (ritonavir‐boosted lopinavir or ritonavir‐boosted atazanavir for children aged 2 to <12 years; ritonavir‐boosted lopinavir, ritonavir‐boosted darunavir, or cobicistat‐boosted darunavir for children aged 6 to <12 years; ritonavir‐boosted lopinavir or efavirenz for adolescents aged 12 to <18 years); FU, follow‐up; Gr 3/4 AE, grade 3 or 4 adverse event.

Figure 3 shows the proportion of participants with safety outcomes reported (all‐cause mortality; grade 3/4 adverse events: clinical, laboratory, both and drug‐related; and discontinuations due to adverse events of any grade). For each study, data are presented separately for each sub‐population (different age groups) and the time point at which events were reported. Mortality was reported in at least one sub‐group within all four studies (Figure 3); there was one death and the cause was accidental [26].

Three studies reported data on grade 3/4 clinical and laboratory adverse events in five sub‐groups of treatment‐experienced children and/or adolescents (GS‐US‐292‐0106, GS‐US‐380‐1474 and GS‐US‐311‐1269) [23, 24, 28, 32]. No events were reported in two sub‐groups of children on F/TAF with boosted protease inhibitor in one study (*n* = 3 and *n* = 9) at 48 weeks (GS‐US‐311‐1269) [32], while events were reported in 25% (3/12) of children on B/F/TAF after 24 weeks (GS‐US‐380‐1474) [28], and in 4% (1/27) and 27% (14/52) of children on EVG/COBI/FTC/TAF after 48 weeks (GS‐US‐292‐0106) (Figure 3) [23, 24]; all were laboratory events (mostly low neutrophils and haematuria). Grade 3/4 adverse events were also reported in 8% (4/50) of ART‐naïve adolescents on EVG/COBI/FTC/TAF over 48 weeks of follow‐up (all clinical events) (GS‐US‐292‐0106) [21]. In two additional groups of children and/or adolescents, data were provided only on grade 3/4 clinical adverse events, and not on the total number of grade 3/4 laboratory events [27, 31]. One grade 3/4 clinical adverse event was reported among 100 (1%) children and adolescents on B/F/TAF in GS‐US‐380‐1474 at 48 weeks follow‐up [26, 27]. The most common laboratory event in this study was haematuria, which occurred in 11 participants (10 of whom were adolescent females with normal menses). In adolescents treated with F/TAF combined with efavirenz or ritonavir‐boosted lopinavir for 24 weeks in GS‐US‐311‐1269, 3/28 participants (10.7%) experienced a grade 3/4 clinical event (gastrointestinal disorders [*n* = 2] and syncope [*n* = 1]) [31]. Grade 3/4 laboratory abnormalities occurring in more than one participant were reported in this study and included amylase (*n* = 5, 18%), fasting low‐density lipoprotein (*n* = 2, 7%) and haematuria (*n* = 2, 7%, all in female participants with normal menses). In both studies, the total number of participants with grade 3/4 adverse events was not reported.

All four studies reported data on drug‐related grade 3/4 adverse events (Figure 3). One event was considered related to TAF: a case of intermediate uveitis in an ART‐naïve adolescent on EVG/COBI/FTC/TAF that resolved without treatment interruption (GS‐US‐292‐0106) [21]. No other drug‐related adverse events were reported.

All four trials reported on treatment discontinuations due to adverse events of any grade. In GS‐US‐380‐1474 (B/F/TAF), there was one discontinuation for an adverse event (grade 2 anxiety and insomnia) out of 100 children and adolescents (1%) over 48 weeks [27]. In GS‐US‐292‐1515 (EVG/COBI/FTC/TAF), two of 50 adolescents (4%) discontinued treatment due to adverse events over the same follow‐up period (one grade 1 iridocyclitis, considered drug‐related; one grade 1 pulmonary tuberculosis, considered unrelated to treatment) [26]. It was not clear in either study if there were discontinuations for other reasons. There were no discontinuations among 28 adolescents during 24 weeks of follow‐up in GS‐US‐311‐1269 (F/TAF with efavirenz or ritonavir‐boosted lopinavir) [31], or among the 12 children followed up for 48 weeks (F/TAF with boosted protease inhibitor) [32]. Likewise, there were no discontinuations by 48 weeks among the ART‐naïve adolescents (*n* = 50) [21] or among the treatment‐experienced children on EVG/COBI/FTC/TAF followed up for 48 weeks (*n* = 27 aged ≥2 years; *n* = 52 aged 6 to <12 years) and 96 weeks (*n* = 19 aged 6 to <12 years) [23, 24].

We were not able to determine if there were differences in safety outcomes between boosted and unboosted regimens as the number of events was small. None of the studies reported on hypersensitivity reactions.

#### 3.1.6 Growth and weight gain

Data on growth and weight gain were collected in all four identified TAF studies and reported in a pooled analysis (*n* = 223): mean height‐for‐age *z*‐scores (HAZ) after 48 weeks of treatment was −0.77, and mean change from baseline was 0.01 (SD not reported) [33]. The mean weight‐for‐age *z*‐score (WAZ) after 48 weeks was −0.2, and the mean change from baseline was 0.25. The proportion of participants who were overweight or obese increased from baseline (overweight, 7%, 16/223; obese, 6%, 13/223) to 48 weeks (overweight, 13%, 29/223; obese, 10%, 22/223). Data for specific sub‐populations were also reported separately for the GS‐US‐292‐0106 study [21]. In treatment‐naïve adolescents on EVG/COBI/FTC/TAF for 48 weeks, there were no notable changes in HAZ from baseline [21]. Among treatment‐experienced children ≥2 years of age in the same study, median HAZ was –0.44 (IQR −1.72, −0.05) at week 24 and −0.51 (IQR not reported) at week 48 (*n* = 27 at both time points) [24]. The change from baseline at these time points was −0.1 (IQR −0.32, 0.23) and −0.001 (IQR −0.42, 0.32), respectively. Median change from baseline in WAZ was 0.04 (IQR −0.17, 0.28) after 24 weeks of treatment, increasing to 0.23 (IQR 0.01, 0.45) at 48 weeks (*n* = 27 at both time points) [24]. Results were not stratified by whether regimens were boosted or unboosted.

Although some information on lipid levels was reported in the pooled analysis [33], only categorical data stratified by whether participants shifted weight category were provided; no absolute levels or change in lipid levels from baseline were reported, which were the endpoints specified in this review.

#### 3.1.7 Bone health

Data on fractures and BMD values for spine and total body less head (TBLH) were provided in three studies [21, 23, 24, 26, 31, 32]. Median percentage change in BMD from baseline and change in BMD *z*‐score (height and age‐adjusted) from baseline are presented in Table 3. One study, GS‐US‐292‐1515, reported a fracture [26]. The grade 1 fracture occurred in an adolescent receiving EVG/COBI/FTC/TAF and was considered unrelated to the study drug.

**Table 3:** Bone mineral density values for spine and total body less head (TBLH) in treatment‐naïve and ‐experienced children and adolescents on tenofovir alafenamide

	Study refs				Follow‐up	Median percent change from baseline (IQR)		BMD height‐age *z*‐score median change from baseline (IQR)	
Study		Population^a^	Treatment	*N*	(weeks)	Spine	TBLH	Spine	TBLH
GS‐US‐292‐0106	[24]	Children (≥2 years; ≥14 to <25 kg)	EVG/COBI/FTC/TAF	12	24	4.2^b^ (0.7, 6.8)	4.2^b^ (2.1, 5.4)	–	–
	[23]	Children (6 to <12 years; ≥25 kg)	EVG/COBI/FTC/TAF	49^d^	48	3.7 (−)	4.2 (−)	−0.12 (−0.46, 0.17)	−0.08 (−0.36, 0.08)
				24^c^	96	7.1 (−)	4.9 (−)	−0.14 (−0.52, 0.42)	−0.35 (−0.71, −0.09)
	[21]	Adolescents (12 to <18 years; ≥35 kg; ART‐naïve)	EVG/COBI/FTC/TAF	50	24^e^	1.3 (−)	0.3 (−)	−0.05 (−)	−0.14 (−)
				50	48	3.3 (0.8, 7.1)	0.9 (0.5, 2.6)	−0.03 (−0.16, 0.2)	−0.09 (−0.33, 0.07)
GS‐US‐292‐1515	[26]	Adolescents (12 to <18 years; ≥35 kg)	EVG/COBI/FTC/TAF	50	48	3.6^f^ (−)	2.8 (−)	0.05 (−)	0.09 (−)
GS‐US‐311‐1269	[32]	Children (2 to <12 years; 17 to <25 kg)	F/TAF+(LPV/r or ATZ/r)	3	24	1.2 (−3.6, 2.6)	2.1 (−0.2, 5.9)	–	–
				2	48	4.3 (3.5, 5.1)	7.1 (6.6, 7.5)	–	–
	[32]	Children (6 to <12 years; ≥25 kg)	F/TAF+(LPV/r, DRV/r or DRV/c)	7	24	1.8 (0.3, 3.7)	2.3 (−2.2, 7.8)	−0.03 (−0.12, 0.08)	−0.13 (−0.41, 0.12)
				6	48	5.7 (1.0, 10.0)	7.0 (4.0, 11.3)	−0.06 (−0.22, 0.04)	0.07 (−0.11, 0.16)
	[31]	Adolescents (12 to <18 years; ≥35 kg)	F/TAF+(LPV/r or EFV)	28	24	3.6 (−0.8, 5.3)	1.6 (0.5, 4.7)	0.03 (−0.21, 0.26)	0.00 (−0.19, 0.15)

Note: “–” indicates that data were not reported in the study.

Abbreviations: ATZ/r, ritonavir‐boosted atazanavir; B/F/TAF, bictegravir, emtricitabine and tenofovir alafenamide; BMD, bone mineral density; DRV/c, cobicistat‐boosted darunavir; DRV/r, ritonavir‐boosted darunavir; EFV, efavirenz; EVG/COBI/FTC/TAF, elvitegravir, cobicistat, emtricitabine and tenofovir alafenamide; F/TAF, emtricitabine and tenofovir alafenamide; IQR, interquartile range; LPV/r, ritonavir‐boosted lopinavir; refs, references; TBLH, total body less head.

^a^ All participants were treatment‐experienced at the start of TAF unless otherwise specified.

^b^ No participants had ≥4% decline in spine or TBLH BMD.

^c^ *N* with follow‐up at 96 weeks.

^d^ *n* = 49 for spine measurements, *n* = 47 for TBLH measurements.

^e^ Of three participants who had at least a 4% decrease in spine BMD from baseline at week 24, one had a persistent decrease of at least 4% at week 48 and none experienced a TBLH BMD decrease of this magnitude.

^f^ BMD decreases of ≥4% occurred in one participant for spine (which increased at subsequent visits) and none for TBLH by week 48.

In treatment‐experienced children assessed across the studies, the median percentage change in BMD at 48 weeks ranged from 3.7% to 5.7 for spine, and from 4.2% to 7.1% for TBLH (Table 3) [23, 32]. In children, the median BMD height‐age *z*‐score change from baseline at 48 weeks was −0.12 (IQR −0.46, 0.17) and −0.06 (IQR −0.22, 0.04) for spine, and −0.08 (IQR −0.36, 0.08) and 0.07 (IQR −0.11, 0.16) for TBLH in the two studies with data reported [21, 23, 31, 32]. In two studies of treatment‐experienced adolescents, the median percentage change in BMD from baseline was 3.6% for spine at 24 and 48 weeks, and 1.6% and 2.8% for TBLH at the same time points (GS‐US‐292‐1515 and GS‐US‐311‐1269) [26, 31]. Median change in BMD *z*‐score was 0.03 and 0.05 for spine at 24 and 48 weeks, respectively, and 0 and 0.09 for TBLH at the same time points (Table 3). Data on change in BMD by prior TDF exposure status were not reported. In treatment‐naïve adolescents in GS‐US‐292‐0106, the median percentage change in BMD from baseline was 3.3% (IQR 0.8, 7.1) for spine, and 0.9% (IQR 0.5, 2.6) for TBLH after 48 weeks of treatment with EVG/COBI/FTC/TAF. At week 24, three of 50 treatment‐naïve adolescents had a greater than 4% decrease in spine BMD from baseline, but by week 48, this was the case for only one participant [21]. No adolescents experienced a BMD decrease of greater than 4% for TBLH. Median change from baseline in BMD *z*‐score at 48 weeks was −0.03 (IQR −0.16, 0.2) for spine, and −0.09 (IQR, −0.33, 0.07) for TBLH [21]. No data were reported on bone health outcomes in children or adolescents on unboosted TAF‐containing regimens.

#### 3.1.8 Renal health

Data on renal outcomes were reported in all four studies and are shown in Table 4. In treatment‐experienced adolescents, the median change in estimated glomerular filtration rate (eGFR) from baseline was 3 ml/min per 1.73 m^2^ (IQR –13, 12) in 28 adolescents after 24 weeks of treatment with F/TAF and either efavirenz or ritonavir‐boosted lopinavir [31] and −19 ml/min per 1.73 m^2^ (IQR −32, −4) in 50 adolescents after 48 weeks of treatment with EVG/COBI/FTC/TAF [26]. In the 50 treatment‐naïve adolescents receiving EVG/COBI/FTC/TAF for 48 weeks, the median change in eGFR from baseline was −12 ml/min per 1.73 m^2^ [21]. No participant had an eGFR consistently less than 80 ml/min at any point during the study. The same study reported that the median change from baseline in serum creatinine was 0.07 mg/dl (IQR 0.02, 0.15). No graded abnormalities in serum creatinine were reported in the 50 treatment‐naïve adolescents, and no cases of proximal renal tubulopathy were reported.

**Table 4:** Median change in estimated glomerular filtration rate (eGFR) from baseline in treatment‐naïve and ‐experienced children and adolescents on tenofovir alafenamide

Study	Study refs	Population^a^	Treatment	*N* with data	Follow‐up (weeks)	Median change in eGFR from baseline (IQR; ml/min per 1.73 m^2^)
GS‐US‐292‐0106	[24]	Children (≥2 years; ≥14 to <25 kg)	EVG/COBI/FTC/TAF	17	24	11 (1, 27)
				27	48	11 (−15, 20)
	[23]	Children (6 to <12 years; ≥25 kg)	EVG/COBI/FTC/TAF	52	48	0 (−14, 15)
				18	96	−14 (−21, 10)
	[21]	Adolescents (12 to <18 years; ≥35 kg; naïve)	EVG/COBI/FTC/TAF	50	24	−15 (−30, 0)
				50	48	−12^b^ (−)
GS‐US‐292‐1515	[26]	Adolescents (12 to <18 years; ≥35 kg)	EVG/COBI/FTC/TAF	50	48	−19 (−32, −4)
GS‐US‐380‐1474	[28]	Children (≥2 years; 14 to <25 kg)	B/F/TAF	12	24	−19 (−24, −8)
	[27]	Children (6 to <12 years; ≥25 kg) and adolescents (12 to <18 years; ≥35 kg)	B/F/TAF	75	48	−17 (−)
GS‐US‐311‐1269	[32]	Children (2 to <12 years; 17 to <25 kg)	F/TAF + (LPV/r or ATZ/r)	2	24	−20^c^ (−22, −17)
				2	48	−11^c^ (−23, 1)
	[32]	Children (6 to <12 years; ≥25 kg)	F/TAF + (LPV/r, DRV/r or DRV/c)	9	24	2^c^ (−9, 11)
				7	48	10^c^ (−4, 19)
	[31]	Adolescents (12 to <18 years; ≥35 kg)	F/TAF + (EFV or LPV/r)	28	24	3 (−13, 12)

Note: “–” indicates that data were not reported in the study.

Abbreviations: ATZ/r, ritonavir‐boosted atazanavir; B/F/TAF, bictegravir, emtricitabine and tenofovir alafenamide; DRV/c, cobicistat‐boosted darunavir; DRV/r, ritonavir‐boosted darunavir; EFV, efavirenz; eGFR, estimated glomerular filtration rate; EVG/COBI/FTC/TAF, elvitegravir, cobicistat, emtricitabine and tenofovir alafenamide; F/TAF, emtricitabine and tenofovir alafenamide; LPV/r, ritonavir‐boosted lopinavir; refs, references.

^a^ All participants were treatment‐experienced at the start of TAF unless otherwise specified.

^b^ No participant had an eGFR consistently less than 80 ml/min at any point during the study. No graded abnormalities in serum creatinine were reported. No cases of proximal renal tubulopathy occurred.

^c^ Changes in eGFR in children were not considered clinically significant.

Across the treatment arms assessing children, the median change in eGFR after 24 weeks ranged from −20 ml/min per 1.73 m^2^ in two children aged 2 to <12 years on F/TAF with ritonavir‐boosted atazanavir or lopinavir to 11 ml/min per 1.73 m^2^ in 17 children aged ≥2 years on EVG/COBI/FTC/TAF [24, 32]. The median change in eGFR after 48 weeks ranged from −11 ml/min per 1.73 m^2^ in two children aged 2 to <12 years on F/TAF with ritonavir‐boosted atazanavir or lopinavir to 11 ml/min per 1.73 m^2^ in children aged ≥2 years on EVG/COBI/FTC/TAF [24, 32] (Table 4).

### 3.2 Summary of findings and implications for research

To our knowledge, this is the first systematic review assessing the efficacy/effectiveness and safety of TAF in children and adolescents. Four single‐arm trials were included (*N* = 341 participants), as well as a pooled analysis of growth and weight gain in a subset of children and adolescents (*N* = 223) aged 6 to <18 years enrolled in the four trials. All participants were taking TAF as a fixed‐dose combination tablet. The majority of children and adolescents (85%) were treatment‐experienced and virally suppressed at baseline. High levels of viral suppression (≥89%) were reported across the sub‐groups of children and adolescents included in the four studies at 24 and 48 weeks, and in the subset of children followed up to 96 weeks. Few discontinuations and grade 3/4 adverse events were observed, with too few events to determine if there was a difference between boosted and unboosted regimens. Although based on non‐comparative studies and a relatively small number of participants, this summary provides some evidence that TAF‐containing regimens are effective through 24–48 weeks in children and adolescents, with no safety concerns reported over the short‐term (24–48 weeks).

Three of these studies were ongoing at the time of this review, and there were limited data on outcomes beyond 48 weeks (19 children aged 6 to <12 years with 96‐week follow‐up). Some additional results were published after the cut‐off date for this review and included a full paper reporting on GS‐US‐380‐1474 [36], and four conference presentations reporting more complete or longer‐term outcomes (to 96 weeks) from GS‐US‐380‐1474 and GS‐US‐292‐0106 [37, 38, 39, 40]. The results from these publications were consistent with the results from the interim analyses included in our review. These findings are also consistent with studies in adults, with viral suppression reported in 94% of individuals on boosted TAF regimens and 89% of those on unboosted regimens [3]. Phase 3 trials in children and adolescents are underway.

Increases in percentage change in BMD were reported in both spine and TBLH during the 48‐week follow‐up, and BMD height‐age adjusted *z*‐scores remained stable. However, comparative studies with monitoring over extended treatment periods are required to ensure that these early growth and bone health outcomes are maintained, as TAF will likely be given to children and adolescents as part of long‐term therapy during key stages of growth and development. Regarding renal health, some decreases in eGFR were observed in participants on boosted regimens, but these were reported to be in line with the inhibitory effect of cobicistat on the tubular secretion of creatinine [21], and were similar to declines observed in adults exposed to cobicistat [41]. Of note, cobicistat is not a recommended treatment for children living with HIV [8]. In GS‐US‐311‐1269, most children were not receiving cobicistat and the changes in eGFR were considered not clinically significant [32]. Data on weight gain in children and adolescents on TAF were reported in the pooled analysis, with slight increases in WAZ observed by 48 weeks. There was also an increase in the proportion of participants classified as overweight or obese in this population [33]. Continued monitoring of weight gain and associated metabolic outcomes in children and adolescents on TAF is critical in light of findings in adults, showing increased weight gain with TAF compared with other NRTIs [42].

### 3.3 Strengths and limitations

The four studies were single‐arm clinical trials with similar eligibility criteria, and all four reported broadly consistent between‐study outcome data at similar time points, despite the lack of a comparator. However, the similarity in study designs limited the generalizability of the data, with single‐arm studies potentially subject to selection bias, which may account for the favourable virological outcomes observed. In paediatric HIV treatment, it is not uncommon for new products to be used in clinical practice on the basis of safety data arising solely from innovator‐funded studies. Comparative and randomized studies, such as the large CHAPAS‐4 randomized controlled trial, will add substantially to the TAF evidence base and could alter our understanding of the efficacy and safety of TAF. With WHO advising that TAF can be used as an alternative NRTI in children for whom an approved dose is available, it will be important to monitor the effectiveness and safety of TAF‐based regimens in heterogeneous populations in real‐world clinical settings; for example, treatment‐experienced populations who are not virally suppressed at baseline, and ART‐naïve children and adolescents, on whom limited data were available. Longer‐term comparative monitoring of safety indicators that do not present clinically, such as bone and renal outcomes (including tubular function), is also needed. In addition, continued development of age‐appropriate formulations of TAF‐containing regimens and research into their use in infants and children weighing <14 kg is needed, to ensure access to the youngest populations and enable harmonization of regimens across age and weight groups. TAF is currently available as part of once‐daily co‐formulations, which provide advantages in terms of reduced pill burden and improved adherence. However, the currently available associated drugs (bictegravir and emtricitabine, or elvitegravir, cobicistat and emtricitabine) are not recommended as WHO‐preferred nor alternative regimens. F/TAF may be of use in combination with dolutegravir, as a WHO‐recommended alternative first‐line regimen for children [8], and paediatric fixed‐dose combinations of F/TAF alone and F/TAF plus dolutegravir are highlighted as priority formulations for research and development [15].

Three of the four identified studies aimed to assess pharmacokinetics, effectiveness and safety, and sample sizes were, therefore, relatively small (≤100). Furthermore, three studies were ongoing at the time of this review, and viral load was estimated using data from participants with follow‐up at the time of reporting rather than from the total study population. These factors might explain some of the variability in the proportion of participants experiencing adverse outcomes, and may preclude observation of rarer safety events. None of the trials included a comparator, and we were, therefore, unable to comment on the efficacy or safety of TAF compared with TDF, abacavir or other NRTIs. This is a critical limitation, as TAF was developed to address some of the adverse events (renal and bone toxicity) associated with TDF. However, we identified no published data that directly compared the two treatments in children or adolescents, and data on prior TDF exposure were not reported. A comparison with abacavir‐containing regimens would also be of interest to assess issues around drug resistance. In the large ongoing phase 3 trial, CHAPAS‐4 (estimated *N* = 1000), participants are randomized to an NRTI backbone of TAF plus emtricitabine or standard of care (lamivudine plus abacavir or zidovudine), and to a third agent consisting of a ritonavir‐boosted protease inhibitor (lopinavir, atazanavir or darunavir) or dolutegravir. This will allow direct assessment of the efficacy of TAF compared with other WHO‐recommended NRTIs in either boosted or unboosted combinations [34].

Although the risk of bias in three of the studies was unclear, the primary reason for this was a lack of information to enable a full bias assessment, as three of the studies were only reported as conference presentations. However, as mentioned above, even well‐conducted single‐arm trials remain subject to potential biases. A limitation of this systematic review was that only English and French publications were included.

## 4 CONCLUSIONS

Four single‐arm trials were identified in this systematic review, and sample sizes were small (*N*≤100). Although these initial findings on the effectiveness and safety of TAF‐based regimens are reassuring, further comparative and longer‐term studies on the use of TAF in children and adolescents are clearly needed. Outcomes of particular importance for additional studies are weight gain and metabolic changes, which have been noted in adults, and bone and renal outcomes, which may not present clinically. Further investigation of TAF used in boosted and unboosted regimens is also needed. If confirmed in comparative trials and more targeted safety studies, TAF has the potential to take a central role in building an effective and well‐tolerated NRTI backbone for treating children and adolescents living with HIV.

## COMPETING INTERESTS

There are no competing interests.

## AUTHORS’ CONTRIBUTIONS

CLT, JOR, EM, IJC, AJ, MV, JJ, VL, FR and MP contributed to developing the study protocol. JOR conducted the searches. JOR, CLT and EM contributed to the screening of abstracts and full papers, extracted the data from selected studies, and assessed the studies for risk of bias. CLT and JOR drafted the paper. JOR, CLT and HC developed the figures. All authors reviewed the manuscript and approved the final draft.

## FUNDING

This work was supported by the World Health Organization and the International AIDS Society's Collaborative Initiative for Paediatric HIV Education and Research (CIPHER).

## DISCLAIMER

The opinions expressed here are those of the authors and do not necessarily reflect the views of the funder. All authors had full access to all the data in the study, and the corresponding author had final responsibility for the decision to submit for publication.

## Supporting information


**File S1**: Details of data sources, search strategies and data extracted.

**File S2**: Supplementary tables and figures.

## Data Availability

The data extracted for this systematic review are available within the article and its supplementary materials.
