# Supplementary material for: Effectiveness and safety of tenofovir alafenamide in children and adolescents living with HIV: a systematic review
Source: J Int AIDS Soc. 2023 Feb 23;26(2):e26037. doi: 10.1002/jia2.26037 (PMC9950035; doi:10.1002/jia2.26037)
Supplement: Supplementary file 2 — File S2: Supplementary tables and figures. [file JIA2-26-e26037-s002.docx]

**Supporting File 2**

**Table S1.** Study characteristics of clinical trials on tenofovir alafenamide in infants, children and/or adolescents, for which no publications were identified at the time of the searches; found through searches of clinical trial registries, March 2021

| **Trial number (study name and design)** | **Title** | **Treatment** | **Estimated completion date** | **URL** |
| --- | --- | --- | --- | --- |
| ISRCTN22964075 (CHAPAS-4, randomised controlled trial) | Children with HIV in Africa – pharmacokinetics and acceptability of simple second-line antiretroviral regimens (CHAPAS-4 trial): a randomised controlled trial | One third drug from (randomised in a 1:1:1 ratio): Dolutegravir (DTG) once-daily (OD), atazanavir/ritonavir (ATV/r) OD, darunavir/ritonavir (DRV/r) OD, or Standard of care: lopinavir/ritonavir (LPV/r) twice-daily (BD). Together with one nucleoside reverse transcriptase inhibitor (NRTI) backbone from (randomised in a 1:1 ratio): tenofovir-alafenamide (TAF) plus emtricitabine (FTC) OD, or Standard of care: whichever of abacavir (ABC) (OD) or zidovudine (ZDV) (BD) has not been used first-line, plus lamivudine (3TC) | Feb-2023 | <https://trialsearch.who.int/Trial2.aspx?TrialID=ISRCTN22964075> |
| NCT02016924 (GS-US-216-0128, Single-arm, three cohorts) | Study Evaluating Pharmacokinetics (PK), Safety, and Efficacy of Cobicistat-boosted Atazanavir (ATV/co) or Cobicistat-boosted Darunavir (DRV/co) and Emtricitabine/Tenofovir Alafenamide (F/TAF) in HIV-1 Infected, Virologically Suppressed Pediatric Participants | Depending on population: cobicistat with either ATV or DRV plus background regimen (12-18 years), cobicistat and emtricitabine/tenofovir alafenamide 200/25 mg with either ATV or DRV (6-12 years), ≥ 3 years will receive cobicistat and F/TAF 120/15 mg with either ATV or DRV. | Apr-2026 | <https://clinicaltrials.gov/ct2/show/study/NCT02016924> |


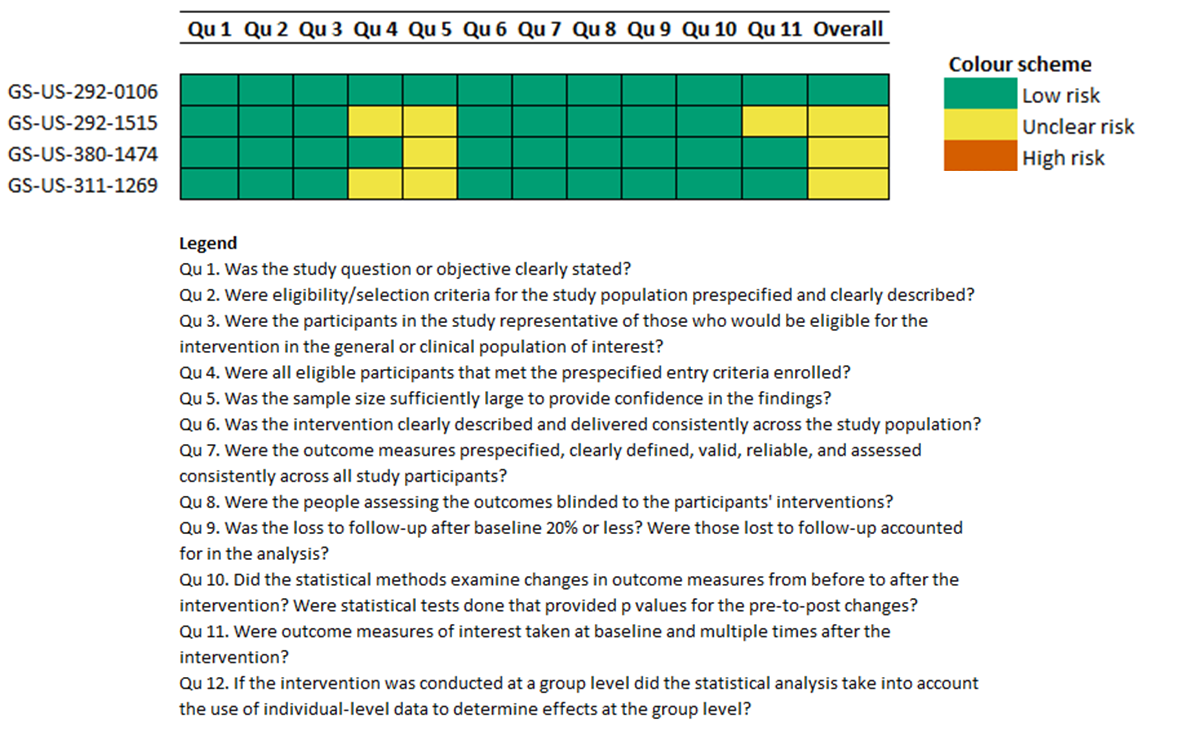


**Figure S1.** Summary of risk of bias assessment using the National Institute of Health quality assessment tool. Green shading indicates low risk of bias, yellow shading indicates unclear risk, and orange shading indicates high risk. Note: None of the interventions were at group level, therefore Question 12 of the risk of bias tool was not applicable and is not presented here.
